# Retinoic Acid-Mediated Control of Energy Metabolism Is Essential for Lung Branching Morphogenesis

**DOI:** 10.3390/ijms25095054

**Published:** 2024-05-06

**Authors:** Hugo Fernandes-Silva, Marco G. Alves, Marcia R. Garcez, Jorge Correia-Pinto, Pedro F. Oliveira, Catarina C. F. Homem, Rute S. Moura

**Affiliations:** 1 Life and Health Sciences Research Institute (ICVS), School of Medicine, University of Minho, 4710-057 Braga, Portugal; hugomiguelfsilva@gmail.com (H.F.-S.); jcp@med.uminho.pt (J.C.-P.); 2 ICVS/3B’s–PT Government Associate Laboratory, 4710-057 Braga/Guimarães, Portugal; 3 PhDOC PhD Program, ICVS/3B’s, School of Medicine, University of Minho, 4710-057 Braga, Portugal; 4 Institute of Biomedicine (iBiMED), Department of Medical Sciences, University of Aveiro, 3810-193 Aveiro, Portugal; alvesmarc@gmail.com; 5 iNOVA4Health, NOVA Medical School/Faculdade de Ciências Médicas (NMS/FCM), Universidade Nova de Lisboa, 1449-011 Lisbon, Portugal; marcia.garcez@nms.unl.pt (M.R.G.); catarina.homem@nms.unl.pt (C.C.F.H.); 6 Graduate Program in Areas of Basic and Applied Biology (GABBA), Universidade do Porto, 4050-313 Porto, Portugal; 7 Department of Pediatric Surgery, Hospital of Braga, 4710-243 Braga, Portugal; 8 LAQV-REQUIMTE & Department of Chemistry, University of Aveiro, 3810-193 Aveiro, Portugal; pfobox@gmail.com

**Keywords:** respiratory system, glycolysis, pyruvate, mitochondria, chicken embryo, cystic lung disease

## Abstract

Lung branching morphogenesis relies on intricate epithelial–mesenchymal interactions and signaling networks. Still, the interplay between signaling and energy metabolism in shaping embryonic lung development remains unexplored. Retinoic acid (RA) signaling influences lung proximal–distal patterning and branching morphogenesis, but its role as a metabolic modulator is unknown. Hence, this study investigates how RA signaling affects the metabolic profile of lung branching. We performed ex vivo lung explant culture of embryonic chicken lungs treated with DMSO, 1 µM RA, or 10 µM BMS493. Extracellular metabolite consumption/production was evaluated by using ^1^H-NMR spectroscopy. Mitochondrial respiration and biogenesis were also analyzed. Proliferation was assessed using an EdU-based assay. The expression of crucial metabolic/signaling components was examined through Western blot, qPCR, and in situ hybridization. RA signaling stimulation redirects glucose towards pyruvate and succinate production rather than to alanine or lactate. Inhibition of RA signaling reduces lung branching, resulting in a cystic-like phenotype while promoting mitochondrial function. Here, RA signaling emerges as a regulator of tissue proliferation and lactate dehydrogenase expression. Furthermore, RA governs fatty acid metabolism through an AMPK-dependent mechanism. These findings underscore RA’s pivotal role in shaping lung metabolism during branching morphogenesis, contributing to our understanding of lung development and cystic-related lung disorders.

## 1. Introduction

Pulmonary branches are formed during the early stages of embryonic lung development through an intricate process known as lung branching morphogenesis. Lung branching shapes the pulmonary airway conducting system and is characterized by epithelial–mesenchymal interactions mediated by complex signaling [1,2]. Lung branching is a highly regulated process and, when disrupted, leads to congenital malformations such as congenital pulmonary airway malformation (CPAM) [3,4].

In the chicken embryo (*Gallus gallus*), the lung arises from the primitive foregut around day 3 of embryogenesis [5]. The primary bronchus (mesobronchus) grows distally, and the secondary bronchi (buds) sprout laterally into the surrounding mesenchyme [6]. This lateral/monopodial branching is similar to the domain branching subroutine observed during mammalian lung development [7,8]. In addition to their structural similarities, the avian respiratory system shares highly conserved molecular mechanisms with mammals, indicating comparable functions [9]. For example, FGF (Fibroblast Growth Factor), SHH (Sonic Hedgehog), WNT (Wingless-related Integration Site), and RA (retinoic acid) play crucial roles in both chicken and mammalian pulmonary branching morphogenesis [10,11,12,13]. These similarities highlight the embryonic chicken lung as an exceptional model for studying lung organogenesis and particularly the early branching processes.

RA signaling is fundamental for vertebrate embryonic development and is a major player in lung organogenesis [14,15,16]. Intracellularly, RA binds to specific nuclear retinoic acid receptors (RARs), of which there are three subtypes (RARα, RARβ, and RARγ). Then, RARs form a heterodimer with retinoic X receptors (RXRs) that mediate downstream cellular signaling and the transcription of RA target genes [17]. RA modulates multiple aspects of embryonic lung development, specifically proximal–distal tissue patterning and branching morphogenesis [13,18,19].

In addition to intercellular signaling, tissue growth involves energy consumption processes and the synthesis of biomolecules. In the embryonic lung, it has been recently described by our group that chicken lung branching morphogenesis gradually adapts to a glycolytic lactate-based metabolism to sustain the lung’s energetic demands, revealing the importance of metabolic regulation in this phase [20]. In the adult lung, metabolic requirements are achieved mainly through the uptake and catabolism of glucose [21,22,23]. However, how signaling networks and energy metabolism cooperate to shape embryonic lung development remains largely unexplored.

The interaction between cell signaling and energy metabolism is starting to emerge as a key concept for understanding developmental processes and developmental abnormalities. Hence, it is crucial to investigate how metabolism influences cellular and developmental decisions, and how metabolism is dynamically regulated during development [24,25,26,27]. Recent studies have demonstrated that metabolism interacts with conserved signaling pathways during development. For instance, in *Drosophila* wing discs, Notch signaling activates glycolysis and suppresses the Krebs cycle, resembling the Warburg effect. Conversely, absence of Notch signaling leads to a decrease in glycolytic gene expression [28]. The Hedgehog signaling pathway can enhance glycolytic ATP production in *Drosophila* wing discs [29]. During neural tissue development in *Zebrafish*, Notch signaling regulates the expression of glycolysis-related genes in a stage-specific manner [30]. Also, an FGF/Wnt signaling coordinated glycolytic gradient regulates cell motility and controls specification, contributing to presomitic mesoderm development during vertebrate embryo somite formation [31].

Considering the well-known role of RA as a modulator of lung organogenesis, we aimed to investigate how the RA signaling pathway modulates the energy metabolism of early stages of chicken lung development. Our study reveals that RA signaling inhibition decreases lung branching and induces a cystic-like phenotype. At the metabolic level, RA signaling is involved in the regulation of glycolysis and pyruvate metabolism, mitochondrial function, and fatty acid metabolism. Here, we show that RA signaling modulates metabolism during early lung organogenesis and that proper RA levels contribute to an adequate metabolic profile during early lung branching. Overall, this report reveals new insights on the RA signaling–metabolism interaction during embryonic lung development that can contribute to understanding the etiology of cystic-related congenital lung disorders.

## 2. Results

### 2.1. Retinoic Acid Signaling Downregulation Decreases Lung Branching and Induces a Cystic-like Phenotype

RA signaling stimulation triggers an increase in lung branching in mouse, rat, and chicken models [13,32,33]. Conversely, the information regarding RA signaling inhibition on lung branching is scarce, even though it is known that its impairment leads to catastrophic effects on the respiratory system development [18]. BMS493 (BMS) is a pan-RAR inverse agonist used to inhibit RA signaling; it specifically suppresses the RA signaling pathway by establishing associations between the RARs and transcriptional co-repressors, thus blocking RA signaling-dependent transcription [14,34].

To determine the impact of BMS on lung branching, we tested several doses that were selected according to the literature [34,35,36,37]. The dose-dependent inhibition effect of BMS on the RA signaling pathway and lung branching morphogenesis led to the choice of the 10 μM of BMS as the best dose to proceed with the studies (please refer to Figure S1 for further details).

To compare the effect of RA signaling stimulation versus inhibition on lung branching morphogenesis, a 48 h ex vivo lung explant culture was performed using stage b2 lungs (2 secondary buds per bronchus). In vitro lung explants were treated with 1 μM of RA (stimulation) or 10 μM of BMS (inhibition); DMSO, the solvent, was used as the control. Afterward, D0 (0 h) and D2 (48 h) explants were morphometrically analyzed, and the results were represented as a D2/D0 ratio. The RA signaling pathway activation status was assessed by performing in situ hybridization for *rarβ*, a recognized target of this cascade [13,32,38].

**Figure 1:**
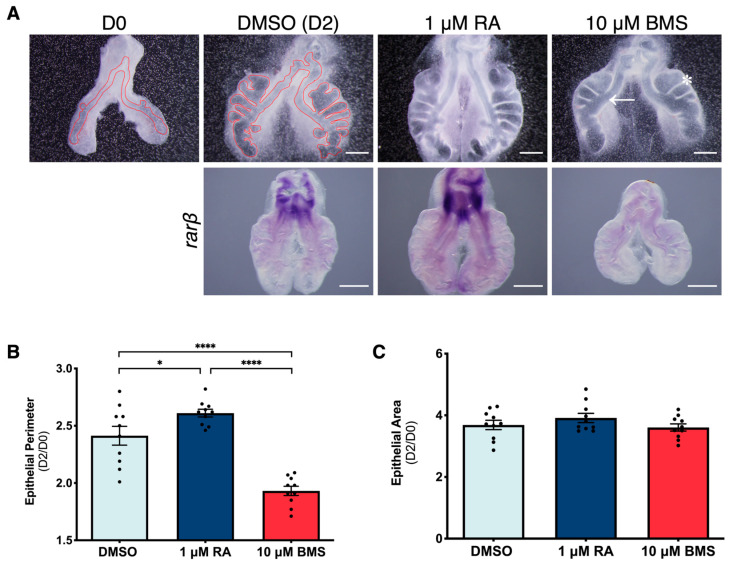
Effect of RA signaling stimulation versus inhibition on lung branching morphogenesis. (**A**) Representative examples of b2 lungs at 0 h (D0) and after 48 h (D2) of explant culture (DMSO, 1 µM of RA or 10 µM of BMS) (upper panel); probed for *rarβ* (lower panel) (*n* ≥ 4/condition). Scale bar: 500 µm. Red line: epithelial compartment outline. Arrow: wider primary bronchus. Asterisk: larger epithelial pouches. The epithelial compartment was outlined (0 h and 48 h DMSO examples) and the epithelial perimeter and area determined. Results are expressed as D2/D0 and represented as mean ± SEM (*n* = 10/condition) for (**B**) epithelial perimeter and (**C**) epithelial area. One-Way ANOVA and Fisher’s LSD tests were performed. Significantly different results are indicated as * *p* < 0.05; **** *p* < 0.0001.

RA treatment increased *rarβ* expression, suggesting an activation of the RA signaling cascade (Figure 1A). On the other hand, BMS treatment decreased *rarβ* expression (Figure 1A), implying a downregulation of the RA pathway. To evaluate branching morphogenesis, the lung’s epithelial compartment was outlined and analyzed, as shown in Figure 1A. There was an increase in the epithelial perimeter of RA-treated lungs compared to DMSO, whereas BMS treatment induced a significant decrease compared to the DMSO (Figure 1B). The changes in the epithelial perimeter were due to alterations in branching morphogenesis since no variations were detected regarding the epithelial area (Figure 1C).

RA is a known stimulator of lung branching morphogenesis, and these results are in agreement with previous RA supplementation studies that have used the embryonic lung [13,32,33]. Furthermore, we show here that BMS treatment downregulates the RA signaling pathway and decreases branching morphogenesis of the embryonic chicken lung. While RA-treated lungs maintained proper branching morphology, BMS-treated lungs displayed a wider primary bronchus (Figure 1A; arrow) and larger epithelial pouches (Figure 1A; asterisk), resembling cystic-like structures. This phenotype is comparable to rat and mouse cystic malformations [3,4].

### 2.2. High Proliferation Is Associated with Active Branching Regions

**Figure 2:**
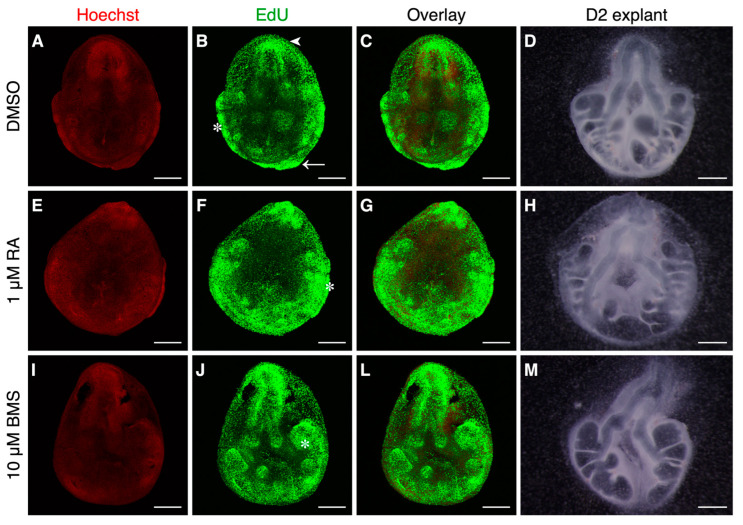
Proliferation analysis of lung branching morphogenesis upon RA signaling stimulation/inhibition. Representative confocal microscopy fluorescence images of b2 lung explants after 48 h in culture (D2), supplemented with (**A**–**D**) DMSO, (**E**–**H**) 1 µM of RA, and (**I**–**M**) 10 µM of BMS. (**A**,**E**,**I**) Nuclei were stained with Hoechst 33342 (Red). (**B**,**F**,**J**) Proliferation assessed through EdU incorporation in DNA, followed by detection using Alexa Fluor 488 (Green). (**C**,**G**,**L**) Merged images. (**D**,**H**,**M**) Lung explants after 48 h of culture and before Hoechst 33,342 staining or EdU incorporation protocols; it is worth mentioning that tissue size reduces after undergoing the proliferation procedure protocol (*n* ≥ 4/condition). Scale bar: 500 µm. Arrowhead: trachea region. Asterisk: active branching sites. Arrow: distal tip.

Considering the effect of RA signaling stimulation vs. inhibition on lung branching morphogenesis, we wondered whether RA signaling modulation could influence the proliferation status of lung branching morphogenesis. For that purpose, we assessed EdU incorporation into new DNA strands using Alexa Fluor 488 (Green) in the three experimental conditions. The nuclei were counterstained with Hoechst 33342 (Red) (Figure 2).

DMSO-treated explants revealed high proliferative areas in the trachea region (arrowhead), in the distal area of the lung (arrow), and at active branching sites (asterisk) (Figure 2B). These results match the previous data described in [20]. Moreover, the proliferation pattern remained unaltered for the 1 µM of RA and 10 µM of BMS conditions (Figure 2B,F,J). Since RA stimulation increases lung branching, and high proliferation is characteristic of active branching sites (asterisk), the regions of high proliferation expand substantially (Figure 2F). In contrast, 10 µM of BMS decreases lung branching morphogenesis and induces a cystic-like phenotype. Thus, in this condition, high proliferation levels are restricted to the branching structures (asterisk) and generally decrease compared to other conditions (Figure 2J).

### 2.3. Retinoic Acid Signaling Stimulation Requires Less Glucose Consumption

**Figure 3:**
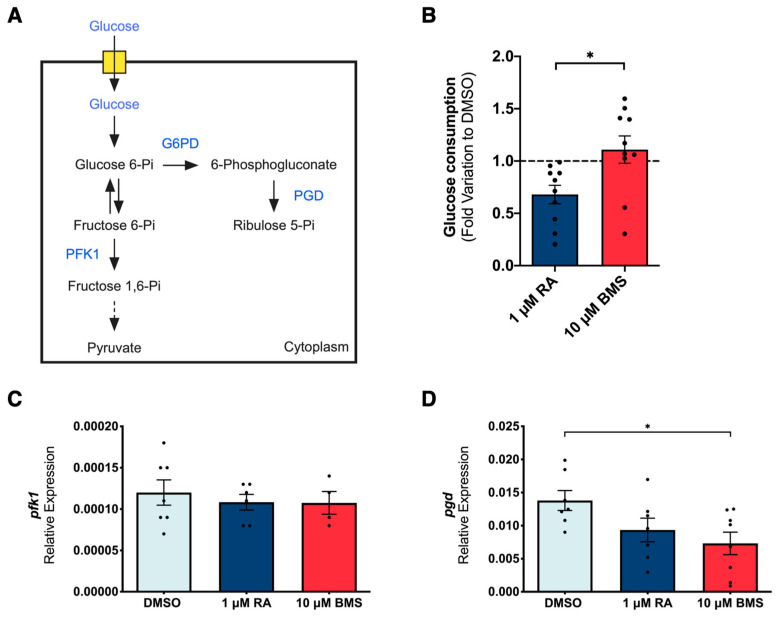
Impact of RA signaling modulation on glucose metabolism during lung branching morphogenesis. (**A**) Schematic representation of glucose metabolism through the glycolytic and pentose phosphate pathways. Blue labeling indicates the molecular targets analyzed; ^1^H-NMR analysis of (**B**) glucose consumption (*n* ≥ 8/condition) during 48 h of lung explant culture (DMSO, 1 µM of RA, or 10 µM of BMS). Metabolite consumption/production was calculated following the mathematical formula |(D1-D0) + (D2-D0)| and represented in fold variation to DMSO. D2 lung mRNA relative expression levels of (**C**) *pfk1* (*n* ≥ 4/condition) and (**D**) *pgd* (*n* ≥ 7/condition). All results are expressed as mean ± SEM. One-Way ANOVA and Fisher’s LSD tests were performed. Significantly different results are indicated as * *p* < 0.05.

Recent data associate intercellular signaling with energy metabolism processes during the tissue growth of embryonic systems [28,29,30,31]. In this sense, we explored potential metabolic alterations induced by RA signaling stimulation versus inhibition. Since both embryonic and adult lungs preferentially use glucose as the primary energy source [20,21,22,23], we started by focusing on glucose metabolism (Figure 3A). For that purpose, lung explants were exposed to one of the following conditions: DMSO, 1 μM of RA, or 10 μM of BMS. The explant culture was performed for 48 h and refreshed at D1 (24 h). The medium was collected at D0 (0 h; reference/control), D1, and D2 and analyzed by using ^1^H-NMR spectroscopy. Extracellular metabolite production/consumption was calculated following the mathematical formula |(D1-D0) + (D2-D0)|, as described in [20], and expressed in fold variation to DMSO. D2 lung tissues were collected to perform qPCR for phosphofructokinase 1 (*pfk1*), glucose-6-phosphate dehydrogenase (*g6pd*), and 6-phosphogluconate dehydrogenase (*pgd*).

The ^1^H-NMR analysis revealed that both RA signaling stimulation and inhibition do not alter lung glucose consumption compared to the control (Figure 3B). Nevertheless, glucose consumption is significantly lower under RA stimulation compared to RA inhibition (Figure 3B). We have previously shown that the embryonic chicken lung has the molecular machinery for the transport and uptake of glucose, and that the glucose consumption profile adjusts to cope with specific energy and nutrient requirements to sustain proper branching morphogenesis [20]. To assess the main enzyme controlling glycolysis [39], we measured *pfk1* expression. *pfk1* expression levels remained unaltered in the three experimental conditions (Figure 3C), meaning that RA signaling modulation does not impact the *pfk1* transcript levels. However, *pfk1* expression displays minimal variations throughout the early stages of lung branching [20].

The main catabolic fate of glucose 6-phosphate is the glycolytic breakdown into pyruvate through glycolysis. However, part of glucose 6-phosphate can be oxidized into pentose phosphates by the pentose phosphate pathway (PPP) (according to the cell needs or NADP+/NADPH concentrations) [39]. To study how RA signaling modulation affects PPP, the expression levels of *g6pd* and *pgd* were evaluated. Both transcripts encode enzymes responsible for the oxidative reactions of PPP that produce NADPH. In the embryonic lung, *g6pd* expression levels are very low, regardless of RA stimulation or inhibition conditions. As for the *pgd*, the expression levels decrease from DMSO to BMS-treated lungs, and RA stimulation displays the same tendency but without statistically significant differences (Figure 3D). According to our results, PPP is not favored under RA signaling stimulation or inhibition, which indicates that glucose 6-phosphate is available to be used by glycolysis. To conclude, under RA signaling stimulation, lung explants seem to adapt to make better use of glucose or to utilize other metabolic substrates, since RA signaling stimulation increases lung branching morphogenesis (Figure 1A,B) with less glucose consumption (Figure 3B).

### 2.4. Retinoic Acid Signaling Controls Pyruvate Metabolism

**Figure 4:**
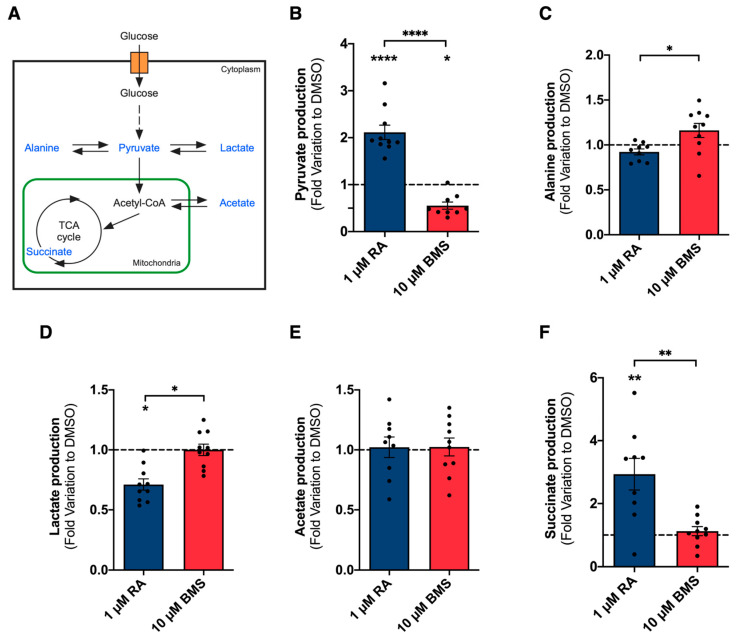
Extracellular metabolite changes induced by RA signaling modulation during lung branching morphogenesis. (**A**) Schematic representation of pyruvate-derived metabolites. Blue labeling indicates the metabolites detected and quantified in the ^1^H-NMR spectra; ^1^H-NMR analysis of (**B**) pyruvate production; (**C**) alanine production, (**D**) lactate production; (**E**) acetate production; and (**F**) succinate production during 48 h of lung explant culture (DMSO, 1 µM of RA, or 10 µM of BMS). Metabolite consumption/production was calculated following the mathematical formula |(D1-D0) + (D2-D0)| and represented in fold variation to DMSO. Results are expressed as mean ± SEM (*n* ≥ 8/condition). One-Way ANOVA and Fisher’s LSD tests were performed. Significantly different results are indicated as * *p* < 0.05; ** *p* < 0.01; **** *p* < 0.0001.

Since glucose consumption between RA signaling stimulation and inhibition was different, we searched for additional glycolysis-related products and further investigated other metabolite alterations promoted by RA signaling modulation. In the ^1^H-NMR spectra, it was possible to detect the final product of glycolysis, pyruvate, and pyruvate-derived metabolites, namely alanine, lactate, acetate, and succinate (Figure 4A).

RA signaling stimulation promotes a sharp increase in pyruvate production compared to the DMSO (≃111% increase) and BMS-treated groups (Figure 4B). In addition, under BMS treatment, pyruvate production is lower than in the DMSO group (≃45% decrease) (Figure 4B). These data suggest that RA signaling stimulation promotes pyruvate production and its accumulation in the extracellular space because it may not be used by mitochondria and may have other metabolic or signaling functions.

No differences were detected in alanine production compared to the DMSO condition; yet, 1 μM of RA produces less alanine than in the 10 μM of BMS-treated group (Figure 4C). This decrease in alanine production is similar to the decrease observed in glucose consumption (Figure 3B).

Furthermore, pyruvate can be inter-converted into lactate through an enzymatic reaction catalyzed by lactate dehydrogenase (LDH), which regenerates NAD^+^ [39]. The conversion of pyruvate to lactate is crucial for lung branching morphogenesis [20]. Moreover, it facilitates the uptake and incorporation of nutrients to form new biomass and sustain active tissue growth [20,40]. Here, we showed that RA signaling stimulation leads to a decrease in lactate production compared to the control and the BMS groups (Figure 4D). This lactate decrease seems to follow the decrease in glucose consumption promoted by RA signaling stimulation (Figure 3B).

After entering the mitochondria, pyruvate is converted into acetyl-CoA, which can be shuttled into acetate or directly fuel the Krebs cycle. Acetate can be used as a substrate for lipid synthesis and might incorporate new cellular membranes to sustain embryonic lung growth [20]. However, no alterations were observed in acetate production among the experimental groups (Figure 4E). Citrate was not detected in the ^1^H-NMR spectra, but succinate production greatly increased upon RA signaling stimulation compared to the control (≃193% increase) and the BMS-treated group (Figure 4F). Succinate is the only direct link between the Krebs cycle and the mitochondrial respiratory chain. During oxidative phosphorylation, the electrons obtained from the succinate/fumarate oxidation through mitochondrial complex II/Succinate dehydrogenase (SDH) are used to reduce ubiquinone to ubiquinol [39]. In addition, the export of succinate from the mitochondrial matrix to the cytosol can act as a signal of mitochondrial status and might contribute to regulating overall metabolic homeostasis [41]. Nevertheless, succinate accumulation can trigger numerous cellular events and act as a metabolic signaling molecule [41,42]. We conclude that upon RA signaling stimulation, there is an increase in the amount of glucose that is directed into pyruvate production rather than into the alanine or lactate branches. In addition, mitochondrial succinate seems to increase at the expense of pyruvate availability.

### 2.5. Retinoic Acid Signaling Modulates Lactate Dehydrogenase Expression

Since RA signaling stimulation promotes a decrease in lactate production during lung branching (Figure 4D), we hypothesized that RA signaling controls lactate dehydrogenase (LDH) expression during branching morphogenesis. To further explore the molecular mechanism underlying lactate production, we analyzed LDHA and LDHB isoforms under RA signaling modulation. The treated explants were processed for in situ hybridization to analyze *ldha* and *ldhb* spatial localizations and relative expression levels. D2 whole lungs were also collected to assess LDHA and LDHB protein expression levels using Western blot.

**Figure 5:**
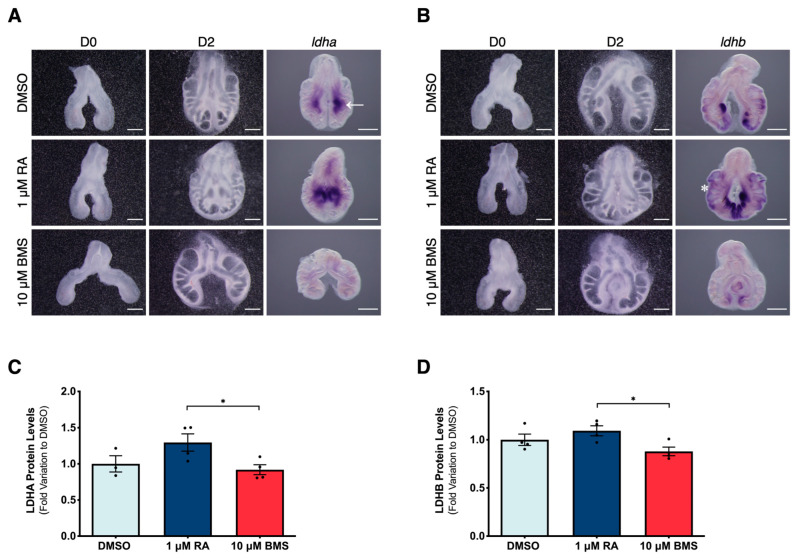
LDHA and LDHB expression alterations induced by RA signaling modulation during lung branching morphogenesis. Representative examples of b2 lung explant culture at D0 and D2, treated with DMSO, 1 µM of RA, and 10 µM of BMS. D2 lungs were probed for (**A**) *ldha* and (**B**) *ldhb* (*n* ≥ 4/condition). Scale bar: 500 µm. Arrow: ventral region. Asterisk: active branching sites; D2 lungs were analyzed for (**C**) LDHA and (**D**) LDHB protein expression levels. LDHA and LDHB immunoblots and total protein are presented in Figure S2. Results are represented in fold variation to DMSO. Results are expressed as mean ± SEM (*n* ≥ 3/condition). One-Way ANOVA and Fisher’s LSD tests were performed. Significantly different results are indicated as * *p* < 0.05.

The *ldha* transcript is limited to the ventral region of the lung (Figure 5A; arrow), and its expression levels are dependent on RA signaling. RA pathway stimulation considerably increases the expression levels of *ldha* compared to DMSO (Figure 5A). Conversely, the BMS treatment decreases *ldha* expression compared to both the DMSO and RA-treated lungs (Figure 5A). Regarding *ldhb*, its expression pattern is restricted to active branching sites (Figure 5B; asterisk). Upon exposure to 1 μM of RA, the expression pattern of *ldhb* is maintained, but its expression levels increase significantly (Figure 5B). On the other hand, lung explants treated with 10 μM of BMS displayed a decrease in *ldhb* expression levels compared to DMSO and RA-exposed lungs (Figure 5B). These results match previous data from our team, wherein both *ldha* and *ldhb* isoforms were present at earlier stages of lung branching and were expressed in a region-specific manner [20]. Moreover, *ldhb* transcript localization was previously associated with active branching regions [20].

LDHA and LDHB protein expression changes are not as pronounced as in the transcripts and this can be explained since Western blot samples comprise whole lungs rather than specific tissue regions (Figure 5C and Figure 5D, respectively). LDHA and LDHB protein levels increase between DMSO and RA-treated lungs, but without statistically significant differences; protein expression levels decrease between RA signaling stimulation and RA signaling inhibition conditions in both isoforms (Figure 5C,D). Here, we describe how LDH expression is modulated by RA signaling and that both *ldha* and *ldhb* isoforms are downstream targets of the RA signaling pathway. Furthermore, high proliferation sites match *ldhb* expression localization when the RA signaling pathway is stimulated (Figure 2B). In this sense, we conclude that high proliferation is associated with active branching regions and that, by modulating lung branching, RA signaling influences the overall lung proliferation rates.

### 2.6. Retinoic Acid Signaling Downregulation Increases Mitochondrial Function

Pyruvate and succinate metabolite data (Figure 4) suggest a potential role of RA signaling in regulating mitochondria function during lung branching morphogenesis. To test this hypothesis, we assessed mitochondrial respiration upon RA signaling stimulation and inhibition. In detail, 48 h lung explant tissues were processed for the real-time measurement of oxygen consumption rate (OCR), and the results were expressed in pmol/min/mg protein (Figure S3).

**Figure 6:**
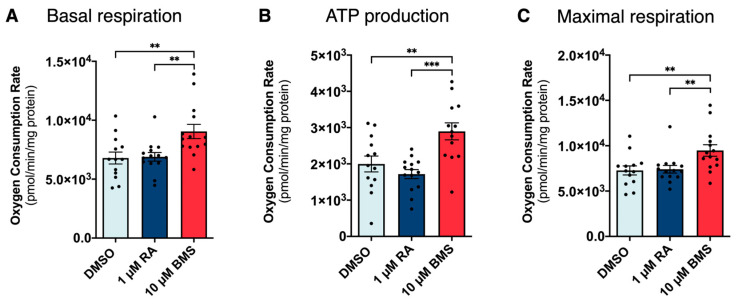
Mitochondrial oxygen consumption of lung explants upon RA signaling stimulation/inhibition. Real-time measurement of oxygen consumption rate (OCR) corresponding to (**A**) basal respiration; (**B**) component of OCR corresponding to ATP production; (**C**) component of OCR corresponding to maximal respiration after 48 h of lung explant culture (DMSO, 1 µM of RA, or 10 µM of BMS). Results are represented in pmol/min and normalized to the total amount of protein. Results are expressed as mean ± SEM (*n* ≥ 13/condition). One-Way ANOVA and Fisher’s LSD tests were performed. Significantly different results are indicated as ** *p* < 0.01; *** *p* < 0.001.

Regarding basal respiration, the OCR from BMS-treated lungs increased compared to the DMSO and RA-treated lungs (Figure 6A). Similarly, for ATP production, the 10 μM of BMS condition displayed higher OCR levels than for DMSO and 1 μM of RA (Figure 6B). Likewise, the maximal respiration component revealed an increase in the OCR levels of the RA inhibition group compared to DMSO and 1 μM of RA (Figure 6C). These results point towards RA signaling stimulation presenting an overall mitochondrial respiration profile similar to DMSO, whereas the BMS-treated lungs display increased mitochondrial function. Overall, these results point to a more oxidative metabolism when RA signaling is inhibited compared to control and RA-treated lungs.

To assess if these differences were due to changes in mitochondrial biogenesis, mtDNA copy number and transcription factor A mitochondrial (*tfam*) expression levels were evaluated by qPCR [43,44]. No differences were observed in mtDNA copy numbers among the three experimental conditions (Figure S4A). Similarly, the expression levels of *tfam* remained unaltered between conditions (Figure S4B). These results suggest the same number of mitochondria among experimental groups, meaning that RA signaling does not affect mitochondrial biogenesis during lung branching morphogenesis.

### 2.7. Retinoic Acid Signaling Controls Fatty Acid Metabolism through AMPK

Considering that RA signaling inhibition promotes mitochondria function and mitochondria plays a pivotal role in lipid metabolism, we decided to investigate how RA signaling modulation impacts lipid metabolism during lung branching. In fact, RA is a known modulator of lipid metabolism [45,46,47]. Moreover, AMP-activated protein kinase (AMPK) is a master regulator of metabolism and a sensor of cellular energy status. AMPK can restore energy balance, modulate glucose and lipid metabolism, and aid mitochondria homeostasis [48,49]. In this sense, we explored whether AMPK and lipid metabolism could be affected by RA signaling modulation during lung branching morphogenesis.

**Figure 7:**
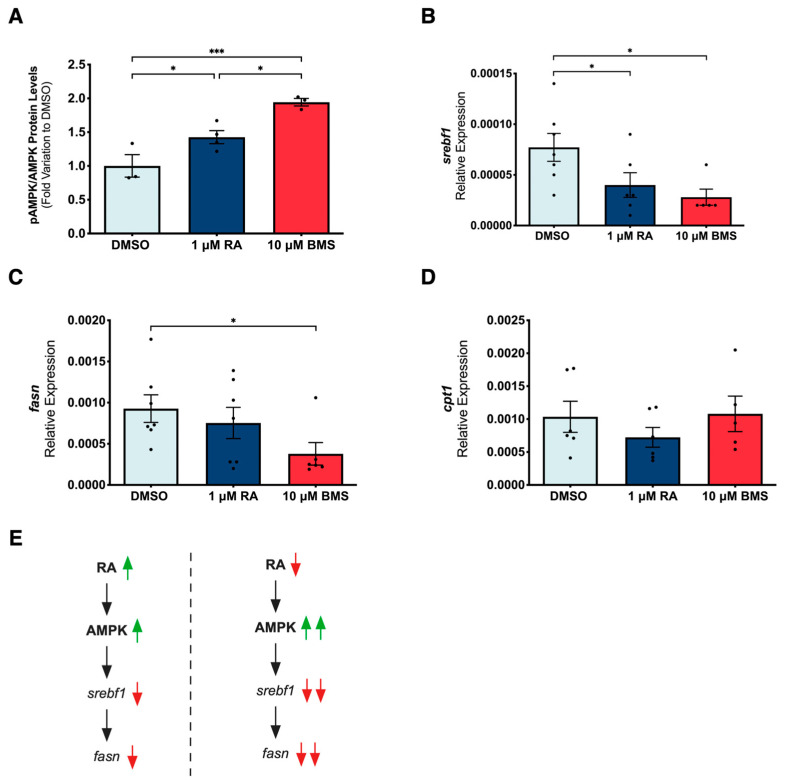
Impact of RA signaling modulation on AMPK pathway and lipid metabolism in early lung branching morphogenesis. D2 lungs were analyzed for (**A**) pAMPK/AMPK protein expression levels (*n* ≥ 3/condition), after 48 h of lung explant culture (DMSO, 1 µM of RA, or 10 µM of BMS). pAMPK and AMPK immunoblots blots and total protein are presented in Figure S5. Results are represented in fold variation to DMSO; mRNA relative expression levels of (**B**) *srebf1*, (**C**) *fasn*, and (**D**) *cpt1* (*n* ≥ 7/condition), after 48 h of lung explant culture (DMSO, 1 µM of RA, or 10 µM of BMS). Results are expressed as mean ± SEM. One-Way ANOVA and Fisher’s LSD tests were performed. Significantly different results are indicated as * *p* < 0.05; *** *p* < 0.001. (**E**) Proposed molecular mechanism involving RA and AMPK signaling pathways, and respective impact on lipid synthesis machinery during lung branching morphogenesis. The symbols refer to increase (↑)**,** decrease (↓), bigger increase (↑↑), or bigger decrease (↓↓).

Our results revealed that RA stimulation upregulated the AMPK pathway through increased pAMPK/AMPK protein expression levels (Figure 7A). Still, under RA signaling inhibition, pAMPK/AMPK protein expression increased even more (Figure 7A). These results suggest that during lung branching morphogenesis, AMPK is activated by RA signaling under stimulation or inhibition conditions. Moreover, the increased activation of AMPK under RA signaling inhibition might be due to a compensatory mechanism used to re-establish tissue homeostasis due to the cystic-like phenotype.

Lipogenesis is, in part, regulated by sterol regulatory element-binding protein 1 (SREBP1). Our results showed that regulatory element-binding transcription factor 1 (*srebf1*) is present in the embryonic lung, with the expression levels decreasing from DMSO to RA-exposed and BMS-treated conditions (Figure 7B). Fatty acid synthase (FAS) is a rate-limiting enzyme of fatty acid synthesis; it converts acetyl-CoA and malonyl-CoA into palmitate [39]. In the chicken lung, fatty acid synthase (*fasn*) expression decreased from the DMSO to BMS-treated lungs (Figure 7C). Together, our data suggest a potential mechanism during early lung branching, in which RA signaling modulation activates the AMPK pathway, which in turn downregulates *srebf1*. The downregulation of *srebf1* decreases the expression of the downstream target *fasn* and, consequently, fatty acid synthesis is reduced.

Fatty acid β-oxidation is the process by which fatty acids are oxidized to acetyl-CoA to produce energy. Carnitine palmitoyltransferase I (CPT1) catalyzes the rate-limiting step of long-chain fatty acid oxidation; it promotes the translocation of fatty acids from the cytosol to the mitochondrial matrix [39]. In the embryonic chicken lung, *cpt1* expression levels remained unaltered between the three experimental conditions (Figure 7D). These data indicate that RA signaling does not modulate fatty acid oxidation during embryonic lung branching.

## 3. Discussion

Growing evidence suggests that signaling–metabolism interactions are essential during animal development [28,30,31]. In this study, we asked whether RA signaling, a well-known modulator of lung organogenesis, could regulate the energy metabolism of lung development during branching morphogenesis.

Our work revealed that RA signaling inhibition, upon BMS treatment, affects lung development by decreasing lung branching morphogenesis and inducing a cystic-like phenotype. This phenotype is comparable to rat overexpression of *fgf10*, which induces cystic malformations similar to human CPAM [4]. Likewise, mouse DICER mutant lungs display an upregulation of mesenchymal *fgf10* accompanied by branching arrest and the formation of large epithelial pouches [3]. An inhibition of FGF10 signaling is also associated with cystic appearances in early pulmonary branching [11]. During lung branching morphogenesis, a gradient of RA is produced from the pleura region to the periepithelial mesenchyme that surrounds the distal region of the growing bud [18]. RA availability regulates *fgf10* expression levels in the mesenchymal compartment surrounding the distal bud tips of the developing lung [11,13,50,51]. Our data indicate that cystic-like structures result from defective RA signaling impairing lung branching. Conversely, and as previously demonstrated, RA signaling stimulation increases lung branching morphogenesis with proper morphology [13,32,33].

After establishing the RA/BMS experimental model, we explored the metabolic alterations induced by RA signaling modulation on lung branching morphogenesis. We took advantage of ex vivo lung explant culture to study the whole organ and precisely assess metabolic and associated molecular alterations in a controlled environment [20,52,53]. Our work revealed that RA signaling stimulation is associated with less glucose consumption than BMS-treated lungs despite increasing lung branching. Thus, RA signaling stimulation promotes a better use of glucose or alternative metabolic substrate utilization. Additionally, other glycolysis-related products were studied, revealing that RA signaling stimulation increases pyruvate production and decreases lactate production in the embryonic lung. Alanine production follows the same tendency as glucose consumption. These findings point to an increase in the amount of glucose directed into pyruvate rather than into alanine or lactate under RA signaling stimulation. Moreover, this also suggests an upregulation of the glycolytic pathway under RA stimulation conditions and without variations in *pfk1* at the transcript level. However, PFK-1 can be modulated by many metabolites, including fructose-2,6-bisphosphate, ATP, AMP, and hormones, potentially suggesting an indirect regulation by RA at the protein/enzyme activity level [39].

Both *ldha* and *ldhb* are downstream targets of the RA signaling cascade. At the protein level, we have noticed the same tendency for LDHA and LDHB but without pronounced differences, which can be explained since we used whole lung tissues rather than isolated tissues from specific regions in the Western blot. Such findings show that RA signaling modulates LDH reaction, namely at active branching sites.

In the ^1^H-NMR spectra, no changes were observed in acetate production, and citrate was not detected. However, there was a significant elevation in succinate levels, which could have had significant effects on various cellular events. This increase in succinate suggests that it may serve as a metabolic signaling molecule, triggering important signaling pathways within the cells [41,42].

In this report, we also showed that proliferation regions are associated with active branching sites and expand substantially upon RA signaling stimulation since this condition increases lung branching. On the contrary, the BMS-treated lungs displayed decreased branching and cystic morphology and were associated with less proliferation overall. Notably, the high proliferation sites match *ldhb* spatial localization upon RA signaling stimulation.

In order to test if RA signaling stimulation promotes pyruvate production to supply mitochondria, we assessed mitochondrial respiration. Curiously, RA signaling stimulation presented an overall respiration profile similar to the control. However, BMS-induced inhibition modulated mitochondria. BMS treatment promoted mitochondrial function, inducing a more oxidative metabolism without influencing mitochondrial biogenesis.

During lung branching morphogenesis, the AMPK pathway is activated by RA signaling under stimulation and inhibition conditions. While AMPK stimulation could be beneficial to a certain extent, in the case of RA signaling stimulation, an overactivation of AMPK by RA signaling inhibition might be related to a compensatory mechanism used to re-establish tissue homeostasis. Similarly, RA inhibits neointimal hyperplasia and suppresses vascular smooth muscle cell proliferation and migration through AMPK signaling activation [54]. Moreover, RA activates the AMPK signaling pathway and sensitizes hepatocellular carcinoma cells to apoptosis induced by sorafenib [55]. RA also activates AMPK in skeletal muscle cells [56]. Our analysis revealed a molecular mechanism in which RA signaling modulation activates the AMPK pathway, downregulating the downstream target *srebf1*. Consequently, the downregulation of *srebf1* decreases the expression of *fasn*, and fatty acid synthesis might be reduced (Fig. 7E). Likewise, in human liver cells, AMPK activation is associated with SREBP-1c inhibition [57]. SREBP influences genes that control epithelial development, proliferation, and cell death, and plays a role in controlling the lung lipid transcriptional network [58]. Moreover, RA treatment inhibits lipid biosynthesis in mice livers [45]. RA signaling does not affect lung branching fatty acid oxidation, which is in contrast to what occurs in other systems [45,46,47].

**Figure 8:**
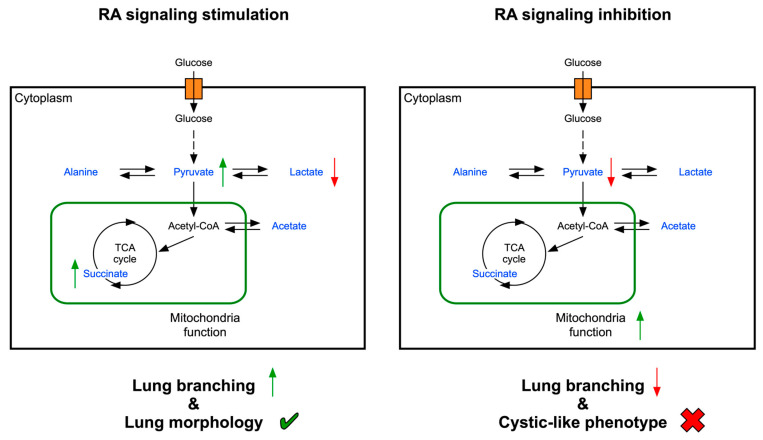
Schematic representation of the metabolic profile of early lung branching morphogenesis upon RA signaling stimulation vs. inhibition. Left panel: Upon RA signaling stimulation there is a metabolic remodeling compared to control lungs. There is a decrease in lactate production and an extracellular accumulation of pyruvate and succinate, which are not used to fuel mitochondria but might exert additional metabolic or signaling roles. In this condition, lung branching morphogenesis is increased and lung tissues display proper lung morphology. Right panel: RA signaling inhibition decreases pyruvate production and increases mitochondrial function, resulting in a more OXPHOS-based metabolic profile. In this condition, lung branching morphogenesis is decreased and lung tissues display a cystic-like phenotype. The symbols refer to increase (↑)**,** decrease (↓), proper lung morphology (✔), or impaired lung morphology (X).

In conclusion, this study describes the metabolic changes produced by RA signaling modulation on lung branching morphogenesis. RA signaling inhibition decreases lung branching and induces a cystic-like phenotype that is accompanied by an increase in mitochondrial function. Since lung branching relies on glycolytic lactate-based metabolism [20], the opposite metabolic profile, the OXPHOS-based BMS-induced metabolism, might explain the observed disease phenotype. On the other hand, RA signaling stimulation increased lung branching while maintaining proper morphology. RA signaling stimulation required less glucose consumption and produced less lactate. Nonetheless, RA stimulation displayed a similar OCR mitochondrial profile as the control lungs. Such data support the extracellular accumulation of pyruvate and succinate, which are not used to fuel OXPHOS. Still, pyruvate and succinate might exert additional metabolic or signaling roles (Figure 8). We hypothesize that RA signaling stimulation might promote optimal growth conditions, while RA signaling inhibition promotes a less efficient metabolism for branching morphogenesis. Moreover, an RA-AMPK-dependent molecular mechanism seems to regulate lipid synthesis in the embryonic lung.

## 4. Materials and Methods

### 4.1. Ethical Statement

Under the European Parliament Directive 2010/63/EU of 22 September 2010 and the Portuguese Directive 113/2013 of 7 August 2013 on the protection of animals used for scientific purposes, no ethical approval was required to carry out this work, which was performed at the early stages of chicken embryonic development.

### 4.2. Tissue Collection

Fertilized chicken eggs, *Gallus gallus*, were incubated between 4.5 and 5.5 days (Embryonic day 4.5–5.5) in an incubator with a 49% humidified atmosphere at 37 °C (Termaks KB400, Fjärås, Sweden). Stage b2 lungs (two secondary buds formed per bronchus) were micro-dissected using a stereomicroscope (Olympus SZX16, Tokyo, Japan) [11]. The dissected lung tissues were processed for ex vivo lung explant culture.

### 4.3. Ex Vivo Lung Explant Culture

Lungs were dissected in PBS and placed on 8 μm nucleopore polycarbonate membranes (Whatman, Marlborough, MA, USA). The lung explants were cultured in 200 μL of medium 199 containing 5.5 mM glucose (Sigma, St Louis, MI, USA),supplemented with 1% (*v*/*v*) L-glutamine (Invitrogen, Waltham, MA, USA), 0.25 mg/mL of ascorbic acid (Sigma), 5% (*v*/*v*) heat-inactivated fetal calf serum (Invitrogen), 10% (*v*/*v*) chick serum (Invitrogen), and 1% (*v*/*v*) penicillin 5000 IU/mL plus streptomycin 5000 IU/mL (Invitrogen). The lung explants were exposed to increasing doses of BMS (BMS493, Sigma): 0.1 μM, 1 μM, or 10 μM; or to a different experimental setting with 1 μM of RA (Sigma) or 10 μM of BMS. DMSO 0.1% was used as the control. The lung explants were incubated for 48 h at 37 °C with 5% CO_2_ (Heraeus HeraCell CO_2_ incubator, Hanau, Germany). At 24 h, the culture medium was replaced by a fresh supplemented medium. The lung explants were photographed at 0 h (D0), 24 h (D1), and 48 h (D2) (Olympus U-LH100HG coupled to Olympus SZX16) and then morphometrically analyzed (AxioVision, Carl Zeiss Microscopy, Oberkochen, Germany). Media samples were collected at D0, D1, and D2 for ^1^H-NMR spectroscopy. D2 tissues were collected for RNA, DNA, and protein extraction. D2 explants were also collected for in situ hybridization, proliferation assay, and seahorse analysis.

### 4.4. RNA Probes

Total RNA was extracted from D2 lung explants using the TripleXtractor directRNA kit (Grisp, Porto, Portugal). Subsequently, 1 μg of RNA was treated with DNase I (Thermo Fisher Scientific, Waltham, MA, USA) and reverse transcribed into cDNA using the GRS cDNA Synthesis kit (Grisp). *rarβ* [59], *ldha*, and *ldhb* [20] RNA probes were produced as previously described. Antisense digoxigenin-labeled RNA probes were produced using T3 (*rarβ*) or SP6 (*ldha* and *ldhb*) RNA polymerase, according to the manufacturer’s instructions (Roche, Mannheim, Germany).

### 4.5. Whole-Mount In Situ Hybridization

After explant culture, the lungs were fixed in PBS solution with 4% formaldehyde and 2 mM EGTA, pH 7.5, at 4 °C overnight. Afterward, the lung explants were dehydrated in a methanol series and stored at −20 °C. The tissues were rehydrated in a methanol/PBT series and processed for whole-mount in situ hybridization (*n* ≥ 4 per gene/condition) [60]. The tissues were permeabilized with proteinase K solution (PBT with 0.05% proteinase K) (Roche). Next, the tissues were incubated in a post-fixing solution (PBT with 10% formaldehyde and 0.4% glutaraldehyde). Subsequently, the tissues were incubated with hybridization solution containing 50% formamide; 6.5% SSC; 1% EDTA, 0.5 M, pH 9.8; 0.5% CHAPS; 0.25% t-RNA; 0.2% heparin; and 0.2% Tween 20; at 70 °C. Then, the tissues were incubated overnight with specific RNA probes in the hybridization solution at 70 °C. The next day, washes were performed with preheated hybridization solution, hybridization solution with MABT (50:50) (5.8% C_4_H_4_O_4_, 4.4% NaCl, 7% NaOH, 1% Tween 20, pH 7.5), and MABT. Next, the tissues were treated with blocking solutions [MABT with 20% blocking reagent (Roche); MABT with 20% blocking reagent plus 20% goat serum (Invitrogen)]. Then, the lungs were incubated in MABT, 20% goat serum, 20% blocking reagent, and anti-digoxigenin antibody (1:2000) (Roche) solution overnight. We committed day 3 to performing MABT solution washes. On the last day, the tissues were washed in NTMT solution (0.1 M Tris-HCl, 0.1 M NaCl, 50 mM MgCl_2_, 1% Tween 20) and then incubated in a developing solution (NTMT with BCIP and NBT) (Roche), protected from light, at 37 °C. The reaction was stopped at the same time for each group of lungs/probes. Lastly, the lung explants were photographed (Olympus U-LH100HG coupled to Olympus SZX16).

### 4.6. ^1^H-NMR Spectroscopy

Explant culture media samples (200 μL) were collected at D0, D1, and D2 and analyzed by using ^1^H-NMR spectroscopy (*n* ≥ 8/condition) according to [61]. Spectra were accessed at 25 °C by using a Bruker Avance 600 MHz spectrometer with a 5 mm QXI probe and z-gradient (Bruker Biospin, Ettlingen, Germany). Solvent-suppressed ^1^H-NMR spectra were acquired with 6 kHz spectral width, 14 s inter-pulse, 3 s water pre-saturation, 45-degree pulse angle, 3.5 s acquisition time, and 128 scans (minimum). Sodium fumarate 10 mM (singlet, at 6.50 ppm) was used as an internal reference. The following metabolites were detected and quantified: H1-α glucose (doublet, 5.22 ppm), pyruvate (singlet, 2.35 ppm), alanine (doublet, 1.46 ppm), lactate (doublet, 1.33 ppm), acetate (singlet, 1.9 ppm), and succinate (singlet, 2.39 ppm). The relative areas of ^1^H-NMR resonances were quantified by using the NUTSpro^TM^ NMR spectral analysis program (Acorn NMR, Livermore, CA, USA). D0 media samples were used as the reference/control. Metabolite consumption or production was calculated using the mathematical formula |(D1-D0) + (D2-D0)| [20] and normalized to the total amount of protein.

### 4.7. Quantitative PCR

Total RNA and cDNA were obtained from stage b2 lungs as previously described. A qPCR method was performed as described in [20]. Specific exon–exon spanning primers were designed for the amplification of the targets (*pfk1*, *g6pd*, *pgd*, *tfam*, *srebf1*, *fasn*, and *cpt1*) and housekeeping transcripts (*18s* and *actin-β*) (Table S1). Primers were optimized for annealing temperature and PCR cycles by using NZY Taq 2x Green Master Mix (NZYTech, Lisboa, Portugal). Afterward, primers were optimized for the efficiency range. Each qPCR was performed in duplicate using 1 μL of cDNA and the SYBR method with the NZY qPCR Green Master Mix (2x) (NZYTech). The *18s* and *actin-β* housekeeping genes were used to normalize the mRNA expression levels. Data on the relative expression levels were calculated using the mathematical model 2^(-ΔCt) [62].

### 4.8. Western Blot

Pooled samples of 10 lungs/pool of D2 lung explants were processed for Western blot as described in [12]. Protein was extracted and quantified according to [63]. Then, 40 μg of protein was loaded onto 10% acrylamide minigels and electrophoresed at 100 V in a Mini-PROTEAN Tetra Cell (Bio-Rad, Hercules, CA, USA). Blotting was performed using low-fluorescence PVDF membranes (Bio-Rad) and a Trans-Blot Turbo Transfer System (Bio-Rad). After that, membranes were incubated with AzureRed Fluorescent Total Protein Stain (Azure Biosystems, Dublin, CA, USA) according to the manufacturer’s instructions. Immunoblots were probed with primary antibodies for LDHA (1:40,000; #3582, Cell Signaling, Danvers, MA, USA), LDHB (1:10,000; #ab240482, Abcam, Cambridge, UK), AMPKα (1:2000; #2532, Cell Signaling), and Phospho-AMPKα (Thr172) (1:2000; #2531, Cell Signaling). Subsequently, the blots were incubated with anti-rabbit IgG HRP-linked secondary antibody (1:5000; #7074, Cell Signaling) or anti-goat secondary IgG (H+L) HRP cross-adsorbed antibody (1:5000; #R-21459, Invitrogen). The membranes were developed with Clarity or Clarity Max Western ECL substrate (Bio-Rad). To capture the chemiluminescent signal, a Sapphire Biomolecular Imager (Azure Biosystems) was used. Western blot quantifications were performed using AzureSpot Analysis Software (Version 2.2.167) (Azure Biosystems) and normalized to the total protein. Two or more independent experiments were performed per pool of tissue (*n* ≥ 3/condition).

### 4.9. Proliferation Assay and Confocal Microscopy

A proliferation assay (*n* ≥ 4/condition) was performed as described in [20]. After 48 h of lung explant culture, half of the explant’s media was replaced by fresh media containing EdU (150 μM final concentration). Explants were incubated with the EdU solution for 90 min at 37°C with 5% CO_2_ (Heraeus HeraCell CO_2_ incubator). Then, the tissues were fixed in PBS with 3.7% formaldehyde. Afterward, the tissues were washed in PBS with 3% BSA and permeabilized in PBS with 0.5% Triton X-100 for 90 min. The issues were washed and processed for the Click-iT Plus EdU reaction according to the manufacturer’s instructions (Click-iT^TM^ Plus EdU Cell Proliferation Kit for Imaging, Invitrogen). Alexa Fluor 488 was used to detect EdU, and the nuclei were counterstained with Hoechst 33342 (1:2000). Image acquisition was performed by using an Olympus LPS Confocal FV3000 microscope (Olympus).

### 4.10. Seahorse Analysis

After 48 h of in vitro lung explant culture, D2 lungs were processed for real-time measurement of oxygen consumption rate (OCR) (*n* ≥ 13/condition) using a Seahorse XFe24 (Agilent, Santa Clara, CA, USA). The seahorse Mito Stress Test was performed according to the manufacturer’s instructions (Agilent, USA). On the previous day, the Seahorse sensor cartridge (Agilent) was hydrated overnight in Seahorse XF calibrant (Agilent) in a non-CO_2_ incubator with humidity at 37 °C. On the protocol day, the Seahorse assay medium was freshly prepared with medium 199 without phenol red (Sigma) and supplemented with 1 mM pyruvate (Sigma) and 1% (*v*/*v*) L-glutamine (Invitrogen), pH 7.3, and warmed at 37 °C until use. Drugs were freshly prepared in the Seahorse assay medium and sequentially loaded into the sensor cartridge injection ports with oligomycin 15 μM (Sigma), FFCP 20 μM (Sigma), and rotenone/antimycinn A 8 μM (Sigma) as the final concentrations. After the explant culture, D2 lungs were washed in PBS and placed on the Seahorse assay media. Islet capture microplates (Agilent) were prepared with 500 μL of the Seahorse assay medium; then, D2 tissues were placed on the wells’ inner depression, and the islet capture screens (Agilent) were placed into the wells. After preparation, both the islet capture microplates (tissues) and the Seahorse sensor cartridge (drugs) were pre-warmed and calibrated at 37 °C. The Seahorse protocol cycles (5;5;5;8) and moment of injections are represented in Figure S3. Each OCR measurement was performed with 3 min of mixing, 2 min of waiting, and 3 min of measuring. In the end, tissues were collected, washed in PBS several times, and processed for protein extraction and quantification. OCR data (pmol/min) were normalized to the total amount of protein.

### 4.11. mtDNA Copy Number

A qPCR method was performed to evaluate the mtDNA copy number as described by [64], with minor modifications. Total DNA was extracted from D2 lungs using a GRS Genomic DNA kit (Grisp). DNA integrity was assessed by using electrophoresis in a 0.6% agarose gel. Specific primers were designed for mitochondrial NADH dehydrogenase 1 (*nd1*) and Nuclear Angiotensin II receptor type 1 (*agrt1*), and the primers were produced according to [65] (Table S1). The primers were optimized for annealing temperature and PCR cycles by using NZY Taq 2×Green Master Mix (NZYTech). Then, the primers were optimized for the efficiency range. qPCR was performed in duplicate (*n* ≥ 8/condition), using 20 ng of DNA per reaction, and the SYBR method was performed by utilizing the NZY qPCR Green Master Mix (2x) (NZYTech). Ct value differences between *nd1* and *agrt1* were used to quantify the mtDNA copy number using the mathematical model 2^(-ΔCt) [62].

### 4.12. Statistical Analysis

Statistical analysis was performed using GraphPad Prism 8 (GraphPad Software, Boston, MA, USA). The normality of distribution was tested using the Kolmogorov–Smirnov test. One-Way ANOVA was performed and followed by a post hoc Fisher’s Least Significant Difference (LSD) test for multiple comparisons. All the data are presented as mean ± standard error of the mean (SEM) with a statistical significance level of 5% (*p* < 0.05).

## 5. Conclusions

RA signaling modulation induces metabolic alterations at the transcript, protein, and metabolite levels. This report unveils novel insights into the signaling–metabolism interaction during embryonic lung development and highlights the importance of metabolic regulation in this phase. Furthermore, our data may contribute to understanding the etiology of congenital lung disorders, namely cystic-related disorders. This is a new and unexplored topic, and several questions requiring additional mechanistic understanding have been raised. Still, this study lays the groundwork for future studies in this emerging field.

## Data Availability

Data are contained within the article.

## Supplementary Materials

The following supporting information can be downloaded at https://www.mdpi.com/article/10.3390/ijms25095054/s1, Figure S1: Dose-dependent effect of BMS treatment on RA signaling pathway and lung branching morphogenesis; Figure S2: LDHA and LDHB full-length blots and total protein; Figure S3: Seahorse OCR profile; Figure S4: Mitochondrial biogenesis in lung branching morphogenesis; Figure S5: AMPK and pAMPK full-length blots and total protein; Table S1: Primers and qPCR conditions.
