# Supplementary material for: Retinoic Acid-Mediated Control of Energy Metabolism Is Essential for Lung Branching Morphogenesis
Source: Int J Mol Sci. 2024 May 6;25(9):5054. doi: 10.3390/ijms25095054 (PMC11084425; doi:10.3390/ijms25095054)
Supplement: Supplementary file 1 [file ijms-25-05054-s001.zip › ijms-2963174-supplementary.pdf]

# Supplementary Materials

## Figure S1

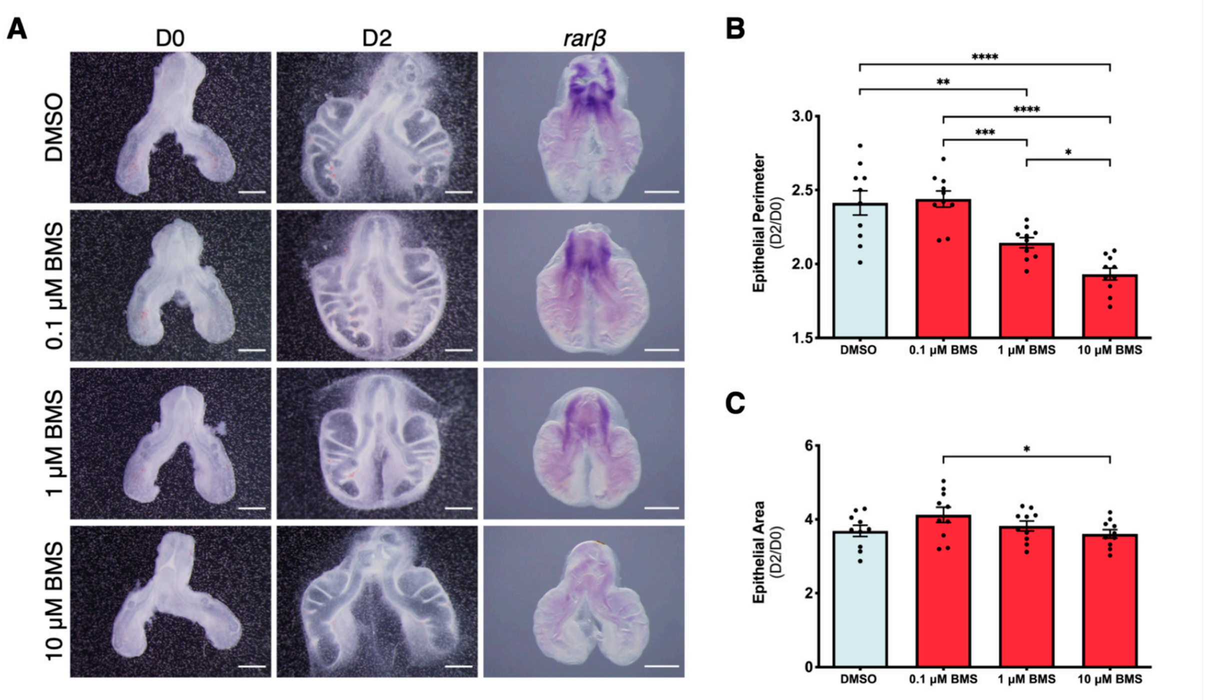


**Figure S1. Dose-dependent effect of BMS treatment on RA signaling pathway and lung branching morphogenesis. (A)** Representative examples of b2 lung explant culture at D0 (0 hours) and D2 (48 hours), treated with DMSO, 0.1 µM of BMS, 1 µM of BMS, and 10 µM of BMS; D2 lungs were probed for *rarβ*, a recognized target of the RA signaling pathway; $n = 5$/condition). scale bar: 500 µm. There is a dose-dependent decrease in *rarβ* expression, suggesting a gradual downregulation of the RA signaling pathway. Morphometric analysis of lung explants: **(B)** epithelial perimeter and **(C)** epithelial area. Results are expressed as D2/D0 ratio and represented as mean ± SEM ($n = 10$/condition). One-Way ANOVA and Fisher's LSD test were performed. Significantly different results are indicated as: *$p < 0.05$; **$p < 0.01$; ***$p < 0.001$; ****$p < 0.0001$. There is a progressive decrease in the epithelial perimeter, and no major alterations were detected in the epithelial area.

**Figure S2**

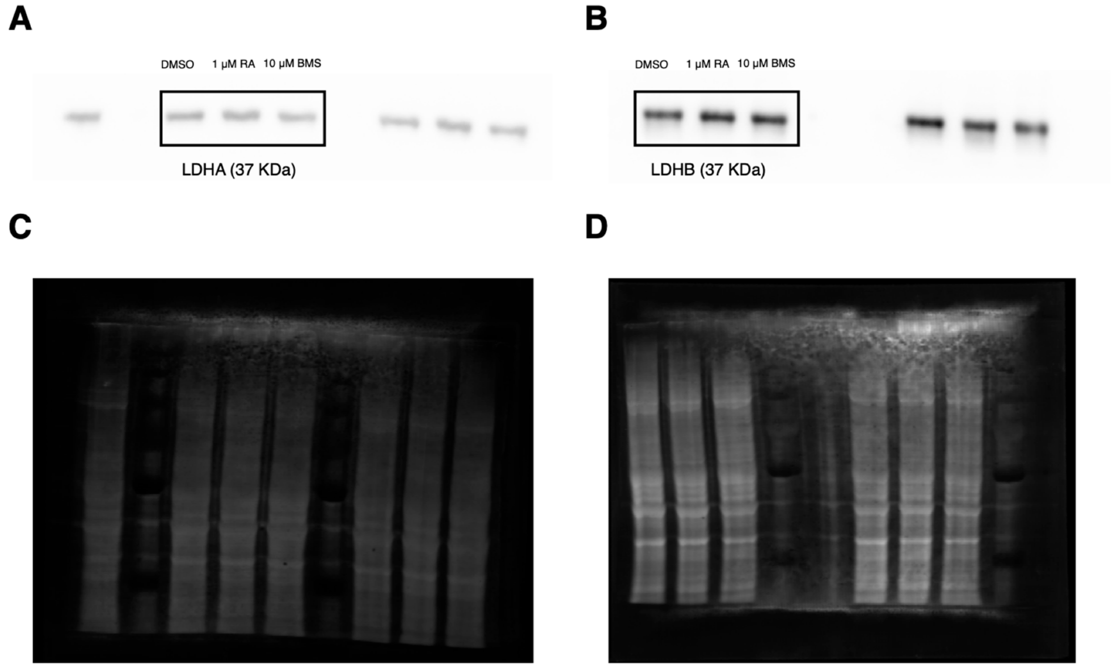


**Figure S2. LDHA and LDHB full-length blots and total protein. (A)** LDHA immunoblot (37 KDa). **(B)** LDHB immunoblot (37 KDa). **(C)** LDHA membrane total protein staining. **(D)** LDHB membrane total protein staining. Quantitative fluorescent total protein stain was performed using AzureRed technology.

## Figure S3

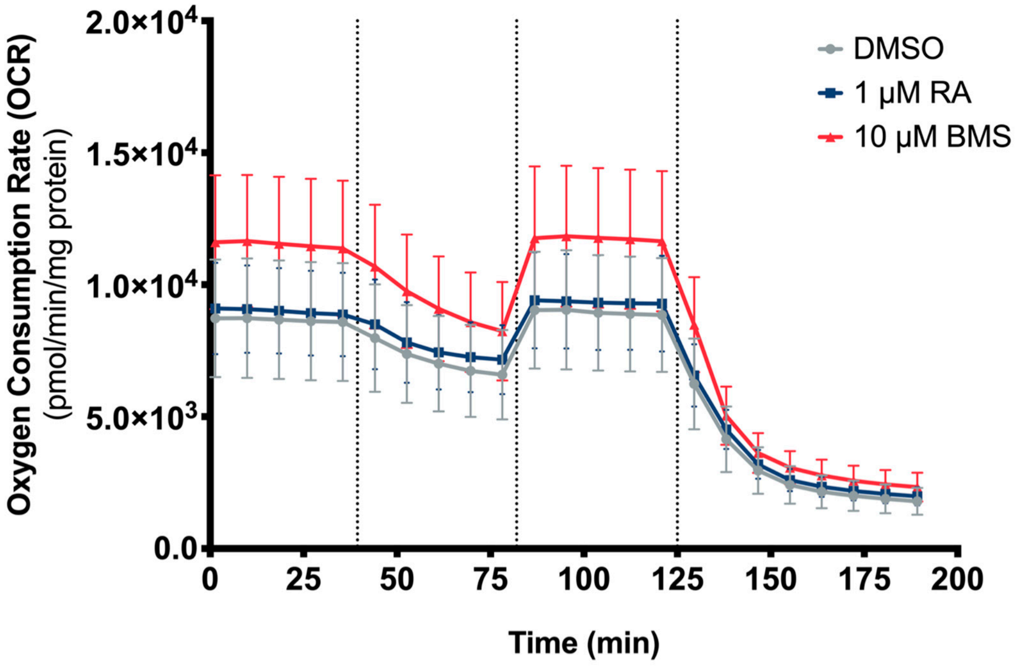


**Figure S3. Seahorse OCR profile.** Real-time measurement of oxygen consumption rate (OCR) of D2 lung explants exposed to DMSO, 1 μM of RA, and 10 μM of BMS. Time of measurements and moment of injections: 1$^{st}$ injection composed of Oligomycin (inhibition of complex V), 2$^{nd}$ injection composed of FFCP (mitochondrial oxidative phosphorylation uncoupler), and 3$^{rd}$ injection composed of Rotenone and Antimycin A (inhibition of complex I and III, respectively). Results are represented in pmol/min/mg protein. Results are expressed as mean ± SEM ($n \geq 13$/condition).

## Figure S4

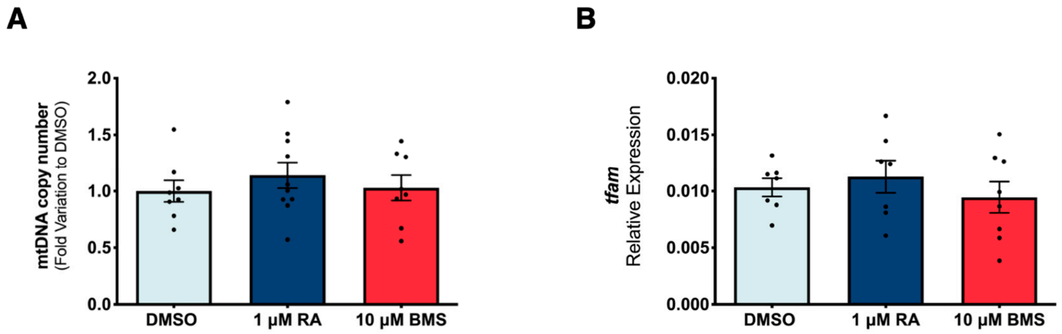


**Figure S4. Mitochondrial biogenesis in lung branching morphogenesis.** Mitochondrial biogenesis analysis of lung explants after 48 hours in culture supplemented with DMSO, 1 µM of RA, and 10 µM of BMS. **(A)** mtDNA copy number ($n \geq 8$/condition). Results are represented in fold variation to DMSO. **(B)** *tfam* relative expression levels ($n \geq 7$/condition). Results are expressed as mean ± SEM. One-Way ANOVA and Fisher's LSD test were performed. No significantly different results.

# Figure S5

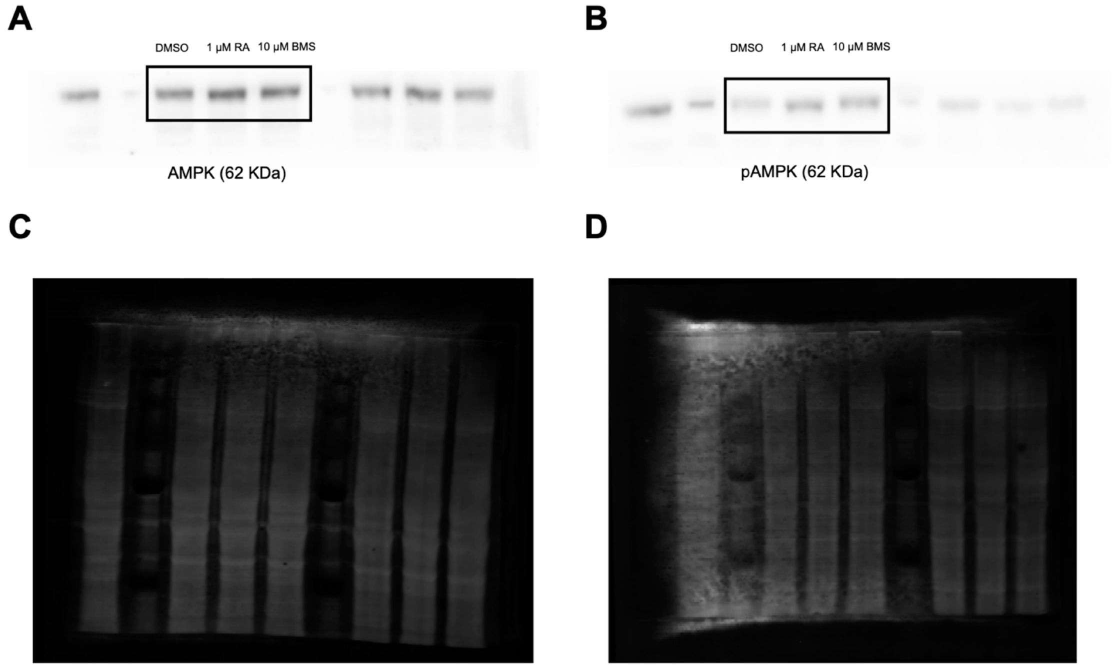


**Figure S5. AMPK and pAMPK full-length blots and total protein. (A)** AMPK immunoblot (62 KDa). **(B)** pAMPK immunoblot (62 KDa). **(C)** AMPK membrane total protein staining. **(D)** pAMPK total protein staining. Quantitative fluorescent total protein stain was performed using AzureRed technology.

## Table S1

**Table S1. Primers and qPCR conditions.** Primer sequences forward (Fw) and reverse (Rv), corresponding PCR product size, annealing temperature and the number of cycles.

| Gene | Sequence 5′-3′ | Size (bp) | Annealing T (°C) | Cycles |
|---|---|---|---|---|
| *actin-β* | Fw - CTTCTAAACCGGACTGTTACCA<br>Rv - AAACAAATAAAGCCATGCCAATCT | 100 | 58 | 30 |
| *18s* | Fw - TCTTTCTCGATTCCGTGGGT<br>Rv - AACGCCACTTGTCCCTCTAC | 157 | 58 | 30 |
| *pfk1* | Fw - CGTGGGAGGAGCTTTGAGAA<br>Rv - CAGCCCACCTCACGTATCTG | 236 | 56 | 40 |
| *g6pd* | Fw - CTGGGGCAGTACGTGGGTAA<br>Rv - CCGAAATATCGCCCGGAACC | 232 | 62 | 40 |
| *pgd* | Fw - AATAAATTAGTGCCGTTGTTGGA<br>Rv - TGGCATGAGTGAAGGACCAT | 174 | 60 | 40 |
| *tfam* | Fw - GGAGAAAACGGCTGGCAAAA<br>Rv - AGCTGAAGGTATGGCTGCTT | 211 | 60 | 40 |
| *srebf1* | Fw - GCTCTCGGCTTCGACGAT<br>Rv - CGAACAGCCCTGAGAAGTCAT | 145 | 60 | 40 |
| *fasn* | Fw - CGGATCTCTCCCACTCTGGA<br>Rv - CCGTGCAATGCCATCTTAGC | 143 | 60 | 40 |
| *cpt1* | Fw - TCATTGCGGGGGAAAACCTC<br>Rv - CCCCACGGCCTTTATTGCTC | 158 | 64 | 40 |
| *nd1* | Fw - TGTAGAATATGCCGCCGGAC<br>Rv - GTCATAGCGGAACCGTGGAT | 214 | 62 | 40 |
| *agrt1* | Fw -TGGCCATAGTGCATCCAGTG<br>Rv - ACGATGAATGATGACGGGCA | 119 | 62 | 40 |
